# Birds of a Feather Resist Together: Sociality and Species Predict the Resilience and Recovery Strategies of Two Neotropical Birds

**DOI:** 10.1002/ece3.71668

**Published:** 2025-06-27

**Authors:** Melissa Ardila‐Villamizar, Daniela T. Sandoval, Adriana A. Maldonado‐Chaparro

**Affiliations:** ^1^ Behavioral Ecology and Conservation Research Group Universidad del Rosario Bogota Colombia; ^2^ School of Biodiversity, One Health & Veterinary Medicine, Graham Kerr Building University of Glasgow Glasgow UK; ^3^ Department of Biology, Faculty of Natural Sciences Universidad del Rosario Bogota Colombia; ^4^ Max Planck Institute of Animal Behavior Radolfzell Germany

**Keywords:** behavioral resilience, human induced disturbance, recovery strategies, tropical birds, urbanization

## Abstract

Behavioral resilience—the ability of animals to recover from disturbances—offers a valuable measure of how urban dwellers cope with human‐induced disturbances. In this study, we conducted behavioral trials across six study sites varying in urbanization level in Bogota, Colombia to assess the resilience and behavioral strategy that great thrushes ( *Turdus fuscater* ) and eared doves ( *Zenaida auriculata* ) employed to achieve it (i.e., recovery strategies). During the trials we measured initial escape responses (flight initiation distance or FID, and alert distance or AD), exposed individuals to a simulated disturbance (human running), and subsequently assessed whether, after the disturbance, they resumed foraging and/or changed their behavior along with their displacement to foraging patches. We also examined the influence of ecological factors such as distance to escape cover, microhabitat, urbanization, flock size, and species in both resilience and the recovery strategies of focal individuals. Our results showed that while most individuals were not resilient, sociality significantly enhanced resilience, with birds in larger flocks more likely to habituate to disturbances. Other factors, such as distance to escape cover, urbanization level and microhabitat did not influence the resilience or strategies employed by individuals. While avoidance was the primary recovery strategy, individuals also reduced their responsiveness, increased vigilance, or adopted a wait and see approach in response to the disturbance. These findings underscore the importance of social behavior and behavioral flexibility in shaping the resilience of urban birds to human disturbance.

## Graphical abstract

Our study evaluated the foraging resilience of great thrushes and eared doves in urban environments, focusing on their capacity to recover from disturbances, the behavioral strategies they employed, and the ecological factors influencing resilience and recovery. We found that urban birds were generally not behaviorally resilient, but sociality and species were significant predictors of resilience, with eared doves in larger flocks being more likely to habituate to disturbances. Additionally, while avoidance was the primary recovery strategy, birds also employed hypervigilance, reduced responses, or waited for disturbances to pass, providing insights into how animals adapt to urban environments through behavioral flexibility.

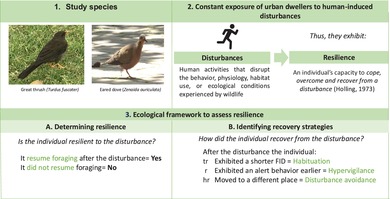

## 1 Introduction

Human‐induced disturbances have negative effects on wildlife as they decrease foraging efficiency and increase mortality (Ciuti et al. 2012), yet some species have successfully colonized urban habitats characterized by high‐intensity disturbances. This suggests the existence of strategies that allow individuals to overcome environmental disturbances, fostering resilience and facilitating their adaptation to such ecological challenges (Chevin et al. 2010). Resilience, defined as the ability of a system (e.g., individual, ecosystem, biological community) to overcome environmental disturbances (Holling 1973) has traditionally been used at the ecosystem level (reviewed by Yi and Jackson 2021). However, recent studies have aimed to assess it at an individual level (e.g., Nattrass and Lusseau 2016; Lisovski et al. 2021). Previous studies have used the resumption of migratory and foraging behaviors as indicators of resilience to understand how individuals overcome environmental changes due to variation in temperature (Nattrass and Lusseau 2016; Lisovski et al. 2021). The resumption of behavior can be a key indicator of resilience, suggesting that an individual regained functionality after a perturbation by either resisting the impact of the disturbance or recovering from it (Morris et al. 2009; Nattrass and Lusseau 2016). However, despite its potential, few studies have empirically used the resumption of behavior as a behavioral metric that indicates resilience.

**FIGURE 1:**
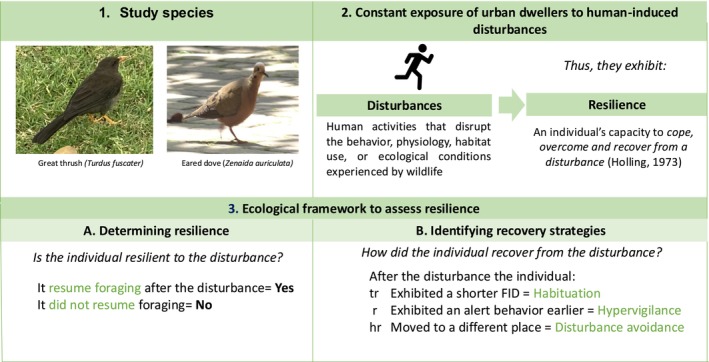
Scheme simplifying the concepts used to assess the resilience. Panel 1 shows the two study species: Great thrush ( *Turdus fuscater* ) and Eared dove ( *Zenaida auriculata* ). Panel 2 shows the definition of two key concepts: Disturbance and resilience. Panel 3 shows the key indicators used to determine the resilience and recovery strategies of the individuals.

The resumption of behavior can be particularly informative within a foraging context since foraging requires animals to continuously balance risk detection with environmental conditions, a principle of the Optimal Foraging Theory (Lima and Dill 1990). To maximize foraging profits, individuals must employ behavioral strategies to cope with disturbances (Frid and Dill 2001) to resume foraging. Escape responses, typically quantified through Flight Initiation Distance (FID)—the distance it takes an individual to escape from a human approaching by foot (Daniel T. Blumstein 2003)—have been widely used to assess the perceived risk during foraging (reviewed by Weston et al. 2012). Hence, comparing the individual's FID before and after experiencing a recurring disturbance allows to identify behavioral changes (suggested by Sol et al. 2013) that reflect how the individual recovered from the disturbance (i.e., recovery strategy): habituation, hypervigilance, or disturbance avoidance. Habituation, characterized by a reduction in FID after experiencing a recurring disturbance suggests perceiving the disturbance as less risky and possible adaptation to it (Daniel Blumstein 2013). Conversely, hypervigilance involves animals to be more cautious toward the disturbance by detecting it earlier (shorter alert distances, AD), which allows them to regulate their response (Fernández‐Juricic et al. 2001). Finally, disturbance avoidance entails animals moving to an undisturbed foraging patch (Dwinnell et al. 2019). Hence, a framework that integrates resumption of behavior as an indicator of resilience, with the comparison of pre‐ and post‐ disturbance escape responses (FID and AD) and displacement as identifiers of recovery strategies, provides a robust method for assessing resilience based on a behavioral metric (Figure 1).

Various ecological factors, such as body size, sociality patterns, and habitat characteristics, such as urbanization, noise levels, and region of the world influence the risk perception of animals and thus their escape responses and displacement after a disturbance (Lowry et al. 2012; Stankowich and Blumstein 2005; Samia et al. 2015; Weston et al. 2021). For example, larger and gregarious animals that inhabit more urbanized and noisier areas or dwell in tropical regions such as African ecosystems tend to perceive less risk from environmental stimuli and thus be more tolerant toward people (i.e., habituate to human‐stimuli) when compared to smaller and solitary individuals that inhabit less urbanized and noisy zones or are present in temperate ecosystems (Lowry et al. 2012; Stankowich and Blumstein 2005; Samia et al. 2015; Weston et al. 2021). As these factors influence the risk perception and displacement of individuals, they are also expected to shape their resilience and recovery strategies, but to our knowledge, this has not been empirically tested yet.

Here, we explored the resilience and recovery strategies of great thrushes (GT) ( *Turdus fuscater* ) and eared doves (ED) ( *Zenaida auriculata* ) when exposed to a human running, a common disturbance in urban ecosystems. We used resumption of behavior as an indicator of resilience and escape responses, displacement, and latency to return as indicators of the recovery strategies. Specifically, to determine the strategies that individuals used to recover from disturbance, we quantified their Flight Initiation Distance (FID), Alert Distance and correct (AD), displacement, and latency to return to the foraging patch during trials. Moreover, we assessed if ecological factors such as distance to escape cover, microhabitat type, urbanization level, flock size and species influenced both individuals' resilience and recovery strategies. We expected individuals to be more resilient to the disturbance when they were foraging: (1) closer to an escape cover as it lowers predation risk (Kullberg and Lafrenz 2007; Tätte et al. 2018), (2) in highly productive microhabitats because the quality of patches can promote foraging rates (Molokwu‐Odozi et al. 2007), (3) in more urbanized areas as individuals there are more exposed to humans and might have acclimatized to them (Lin et al. 2012), (4) in larger flocks because group‐foraging can reduce how individuals experience different ecological pressures such as predation and disturbance (Ydenberg and Dill 1986). Finally, since our study species have different life history traits, we expected them to differ in their resilience and the strategies they employed to recover from disturbance. Specifically, we expected ED to be more resilient and exhibit habituation strategies since they are well acclimated to human presence (Fontoura 2013), whereas GT were expected to be less resilient and exhibit hypervigilance strategies because they are less used to human presence and are a territorial. Territorial species often exhibit heightened vigilance allowing earlier detection of human approaches (Tätte et al. 2019). However, territoriality may have complex effects on resilience as highly territorial individuals are closely tied to their territories (The habitat connectivity hypothesis: Radvan et al. 2023) which may increase their resilience.

## 2 Materials and Methods

### 2.1 Study Area and Species

This study was conducted in Bogota, the largest, most urbanized, and populated city in Colombia, characterized by an heterogenous landscape in terms of urbanization (Cadenasso et al. 2007; Páramo‐Rocha 2003). Within the city, we identified six sites and characterized their vegetation cover extension, type of infrastructure and pedestrian and domestic animal density (following Ardila‐Villamizar et al. 2022). We classified sites into three categories representing the different levels of urbanization that birds experience throughout the city: residential areas, metropolitan parks, and zonal parks (Figure S1). Residential areas were sites with low vegetation cover, high residential infrastructure, and frequent vehicular transit. Metropolitan parks were areas with medium vegetation cover (i.e., less than natural sites within the city but more than residential areas) that restrict the access of domestic animals and are highly visited by the public. Zonal parks also had medium vegetation cover however, there do not have pet restrictions and are less visited than metropolitan areas. We selected study sites based on their accessibility and safety and, to ensure a balanced spatial representation across the city. Finally, we did not include natural areas in this study because recreational activities such as running are restricted there and to avoid disturbing animals in undisturbed areas.

We focused our study on two native species that are predominant throughout Bogota and thus offer a major opportunity to test resilience in urban environments: great thrush and eared dove. GT are a highly territorial species that can be found across the Andean highlands in a variety of habitats, from urban areas to temperate forests (Hilty and Brown 1986). ED can be found in different habitats throughout South America (Fontoura 2013). They a are neophile species that seem to be well adapted to human presence, probably due to their ability to nest and forage on urban resources (Fontoura 2013).

### 2.2 Data Collection

#### 2.2.1 Behavioral Trials

We used resumption of behavior as an indicator of resilience and variation in escape responses, displacement, and latency to return to characterize the recovery strategies. Hence, we designed trials that allowed us to assess (1) if individuals resume foraging after the disturbance (i.e., if they were resilient) and if so, (2) how do they recover from it (i.e., the recovery strategies).

**FIGURE 2:**
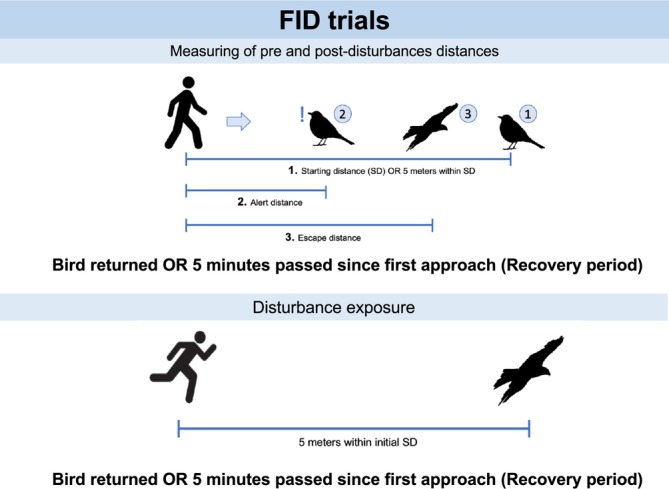
Scheme representing the methodology of the FID trials. Panel on top illustrates the procedure of quantifying the flight initiation distance or FID of focal birds before and after being exposed to a disturbance. Panel bellow shows how we exposed focal birds to a high intensity disturbance (running toward them). All the pictures used in the scheme (human walking, bird on the ground, birds flying and human running) are licensed under Creative Commons. Pictures of the human walking and running were recovered from https://www.clipartmax.com and, of the bird on the ground and flying from https://www.klipartz.com.

Trials consisted of three key steps. First, we measured the pre‐disturbance FID and AD of individuals, which allowed us to assess its baseline escape behavior (see section 2.2.1.1). Second, we exposed individuals to a controlled disturbance (i.e., human approached by running) (see section 2.2.1.2) and identified whether individuals resumed foraging (i.e., were resilient) and the escape responses exhibited after the perturbation (see section 2.2.1.3 and Figure 2). Therefore, in each trial we quantified four behaviors: (1) FID defined as the distance it took an individual to escape from an approaching human (Daniel T. Blumstein 2003), (2) AD, the distance it takes an individual to exhibit an alert behavior when being approached by a human (Fernández‐Juricic et al. 2001), (3) Displacement, the action in which an individual changes its location after being disturbed (Bradbury et al. 2015), and (4) Latency, the time it takes an individual to return to its foraging patch after being disturbed (Jacob and Brown 2000). Later, we used these behaviors to determine the resilience and the strategy that individuals employed to recover from disturbance (See section 2.3).

Each trial was carried out by two researchers, an experimenter (MAV) and an observer (DTS), in six study sites: two residential areas, two metropolitan parks and two zonal parks (Figure S1). At the beginning of the sampling session, both experimenter and observer followed random transects within the study sites searching for focal individuals. An individual was considered as focal if it belonged to one of the study species, had not been previously disturbed by any environmental stimuli (e.g., pedestrians, domestic animals, cars), was foraging on the ground on its own or in a flock and it was within a 10–30 m radius from the researchers (adapted from Geist et al. 2005).

##### 2.2.1.1 Measuring Pre‐Disturbance Escape Distances

Once a focal individual was identified, the observer moved 10 m away and to the right of the experimenter, after which the trial began. First, the experimenter signaled her starting position with a color marker (starting distance) and began to walk toward the focal individual at a constant pace (~0.8 m/s). When the focal individual exhibited an alert behavior, such as raising its neck or directing its gaze at her, the experimenter dropped another color marker (pre‐disturbance AD) and continued the approach without interrupting it until the focal individual exhibited an escape response: walking, hopping, or flying (pre‐disturbance FID). The observer kept track of the bird's position after escaping and measured: (1) the time it remained on the ground after being disturbed when its escape response was walking or hopping (waiting time); or (2) the time it took it to return within 10 m from the site where it was first approached when its escape response was flying (latency time). Whilst the observer noted the waiting and latency time, the experimenter measured the starting, alert, and escape distances through paces or using a laser range finder (BOSCH GLM 20) when possible. In case the bird moved after being approached, the experimenter also quantified the distance between the place the bird was first approached and where it moved after it (pre‐disturbance displacement). Once finished taking the measures, the experimenter moved away from the bird to where the observer was located to avoid influencing the escape behavior or recovery from the disturbance.

##### 2.2.1.2 Disturbance Exposure

The researchers proceeded to expose the focal bird to a disturbance (running toward them) when the focal bird returned to within five meters of its original location (i.e., recovering period), or when 5 min had passed after approaching a focal bird that remained on the ground (i.e., recovering period). We decided to wait up to 5 min to a avoid stressing the focal birds that remained on the ground and because during the pilot trials, we observed that focal birds that moved away were less likely to return after this time. If the focal bird did not return after this time, the trial was concluded. To expose birds to the disturbance, the experimenter located herself within five meters of the distance she initially approached the focal bird and ran toward it at a constant speed (~4.8 m/s). After the bird was disturbed and escaped, the observer kept track of it and quantified its waiting and latency time after the disturbance. In case the bird moved after being ran toward, the experimenter also measured the distance between the place the bird was approached and where it moved after this (disturbance displacement).

##### 2.2.1.3 Measuring of Post‐Disturbance Escape Distances

Once the recovering period was over (i.e., either the bird returned to within 5 m of its original location prior to the initial approach or 5 min had passed since the disturbance), the experimenter moved within five meters of the pre‐disturbance starting distance. We aimed to keep this distance constant because starting the approach from a further distance could influence their FID (Daniel T. Blumstein 2003). Then, the experimenter walked toward the bird once again to measure its post‐disturbance starting, alert, and escape distances.

For each trial we also noted the behavior that the bird exhibited after being approached (i.e., walked, hopped or fled), the microhabitat where the focal bird was located when being approached (grass, bare ground or concrete), and the pre‐ and post‐disturbance distance to the nearest escape cover.

The sampling sessions were conducted from 7 to 10 AM from January 11th to March 3rd of 2024 as often as possible but at least twice a week. The experimenter trained pace calibration to maintain a constant speed whilst approaching and running toward the bird, and identification of escape and alert behavior routinely 1 week before data collection. Lastly, to avoid testing the same bird twice, trials were conducted at least 25 m from each other.

### 2.3 Recovery Strategy

We classified the strategy employed by focal birds based on the following criteria: (1) if the bird returned or not after being disturbed (i.e., behavioral resilience), (2) changes in the alert and escape distance before and after the focal bird was disturbed, and (3) the displacement distance after the disturbance (Table 1). Thus, to classify the strategies, we compared the pre‐ and post‐disturbance FID, AD, and movement of the tested birds.

**TABLE 1:** Classification of the recovery strategies that focal birds employed to recover from disturbances.

Strategy	Returned?	FID after disturbance	AD after disturbance	Movement after disturbance?
Habituation	Yes	<	=	No
Hypervigilance	Yes	=	>	No
Wait and see	Yes	=	=	Yes
Disturbance avoidance	No	NA	NA	Yes

*Note:* Categorization was based on: If the bird returned, changed their FID and AD and if they moved after being disturbed.

We classified recovery strategies in: (1) habituation when after being disturbed, the bird returned or stayed in the ground (i.e., exhibited behavioral resilience), stayed in the same place or within 2 m from it and their post‐disturbance escape response was shorter than their pre‐disturbance escape response; (2) hypervigilance when birds were resilient to the disturbance, did not displace more than 2 m after the disturbance and their post‐disturbance alert response was exhibited earlier than when they were approach before the disturbance; (3) “wait and see” if the individual recovered, did not change their escape and AD after the disturbance but moved within 10 m away from its initial position after being perturbed; and (4) disturbance avoidance if they did not recover from the disturbance and moved more than 10 m after it (Table 1).

### 2.4 Ecological Factors That Might Affect the Resilience and Recovery Strategies of Birds

We quantified six ecological factors that we believed could affect resilience and recovery strategies: noise level, pedestrian density, domestic animal density, urbanization level, sex and flock size. We measured the noise level, pedestrian density and domestic animal density of the study sites at the beginning (7 AM), middle (8:30 AM), and end (10 AM) of each sampling session to generate an average of each variable per sampling day. Each measure was recorded at different sites (for the exact location of each trial see Figure S1). Noise level (dB) was measured using a sound meter (UNI‐T UT353‐BT). Pedestrian density (walkers/min) and domestic animal density (domestic animals/min) were quantified by counting the number of people (for pedestrian density) or dogs (for domestic animal density) that walked by in 15 min (following Mikula 2014). Flock size was measured at each trial as the number of conspecifics that were in a 10‐m radius from the focal individual (following Clucas and Marzluff 2012).

The urbanization level of five of our study sites was obtained from (Ardila‐Villamizar et al. 2022). For the other site (Parque Metropolitano El Tunal) the urbanization level was obtained following the same procedure reported by (Ardila‐Villamizar et al. 2022).

### 2.5 Statistical Analysis

For the analysis, we included only individuals tested at a starting distance of 10–30 m and those for which the difference between pre‐ and post‐disturbance starting distances did not exceed 5 m. All analyses were conducted in R v 4.1.2 (R Core Team 2021).

We selected the variables pre‐disturbance distance to escape cover, urbanization level, flock size, microhabitat, and species as predictors of our models because they have been shown to influence antipredatory behaviors of urban dwellers (Distance to escape cover: (Morelli et al. 2022; Tätte et al. 2018), Flock size: (Ardila‐Villamizar et al. 2022; Morelli et al. 2019), Urbanization level: (Hall et al. 2020; Ardila‐Villamizar et al. 2022), Species: (Møller 2010; Daniel Blumstein 2006)). Thus, we expected them to also influence their resilience and recovery strategies. To our knowledge, there are no studies that have assessed the influence of microhabitat on antipredatory behavior. However, we decided to include it because it can be a proxy of the quality of the foraging patch, which could influence foraging decisions (Shochat et al. 2004) and thus, resilience and recovery strategies.

#### 2.5.1 Which Ecological Factors Influence the Resilience of Urban Dwellers?

To identify the factors that affected the resilience of focal individuals (i.e., if they recovered or not from the disturbance), we fitted a Generalized Linear Model (GLM, *N* = 113) with binomial distribution using the glm function from base R (R Core Team 2021). We fitted resilience (yes = 1/no = 0) as a response variable and pre‐disturbance distance to escape cover, urbanization level, flock size, microhabitat and species as untransformed scaled predictors. We only considered pre‐disturbance escape cover distance but not post‐disturbance values because we did not have post‐disturbance distances data from all the individuals (most of them did not recover from being ran toward; *N* = 82 out of 113). We tested for collinearity among the fitted variables calculating the variance inflation (VIF) of the GLM using the function vif from the “car” package v 3.1‐0 (Fox and Weisberg 2019) and did not find it. We initially tested for study site as a random effect but removed it because its effect null (*V* = 0, SD = 0, *N*_total_ = 113: *N*_residential areas_ = 37, *N*_met parks_ = 39, *N*_zonal_ = 37).

#### 2.5.2 Do Ecological Factors Influence Recovery Strategies?

We performed a Multinomial Logit Model for Categorical and Multinomial Responses to assess if the strategies that individuals employed to recover from disturbance were influenced by ecological factors (urbanization level, microhabitat, species and flock size before being disturbed; *N* = 113). First, we fitted an initial model with the ecological factors as predictors and recovery strategies as the independent variables using the mblogit function from the “mclogit” package v. 0.4.4 (Elff et al. 2022). Then, we checked the collinearity among the model terms using the check_collinearity function from the “performance” package v 0.12.3 (Lüdecke et al. 2021).

We found multicollinearity among the model terms. Microhabitat had a high VIF (VIF_microhabitat_ = 39.12), indicating strong collinearity with other predictors, and was therefore excluded from the model. However, after removing it, VIF values remained high; thus, we also excluded urbanization level (VIF_urbanization level_ = 12.31) from this model. Thus, we performed a Multinomial Logit Model for Categorical and Multinomial Responses fitting only flock size and species as predictors and recovery strategies as an independent variable.

## 3 Results

We tested 117 individuals, 57 GT and 60 ED. We removed 4 observations from our dataset because either their starting distance was longer than 30 m (*N* = 2) or the difference between the pre‐ and post‐disturbance was > 5 m (*N* = 2). Thus, our dataset for all statistical models consisted of 113 measures (GT:57 and ED:56), corresponding to, 37 individuals in residential areas (GT: 17, ED: 20), 37 in zonal parks (GT:20, ED:17) and 39 in metropolitan parks (GT:20, ED:19). 31 of tested individuals remained on the ground or returned after being exposed to the disturbance (i.e., were resilient) (GT: 7 and ED: 24) whilst 82 did not (GT: 50, ED: 32). Generally, individuals were located 4.26 ± 4.72 m from the closest escape cover when first approached and were found foraging by themselves or as part of flocks from two to five individuals (*N*_alone_ = 84, *N*_in flocks_ = 29). Also, on average, individuals experienced 42.07% ± 21.81% of urbanization. Lastly, the recovery strategy that most individuals exhibited was disturbance avoidance followed by low resistance, habituation, hypervigilance and wait and see (Table S1).

### 3.1 Resilience Was Influenced by Flock Size and Varied Among Species

**FIGURE 3:**
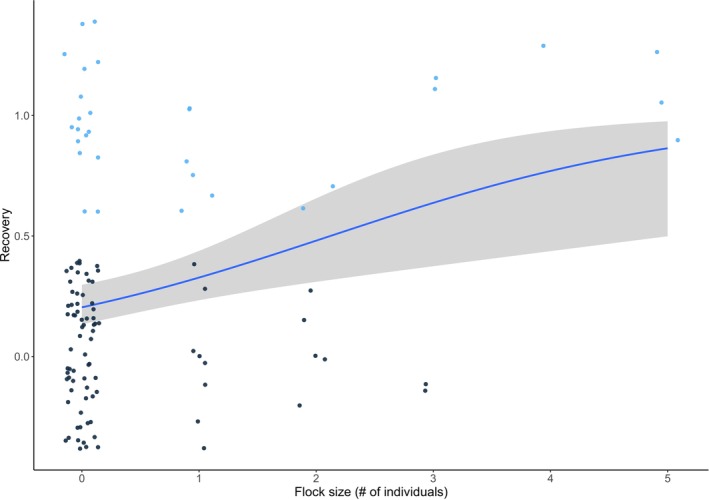
Relationship between recovery (1 = yes, 0 = no) and sociality patterns (*N* = 113). The blue line represents the regression line of the main effects and the shaded gray zone its 95% confidence interval. Dots represent raw data (jittered for readability). Light blue dots represent individuals that recovered and dark blue dots those that did not.

**FIGURE 4:**
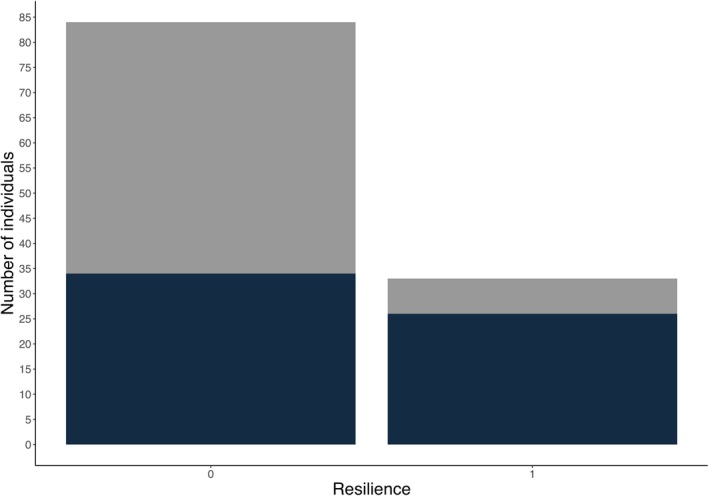
Differences in resilience between great thrushes (represented by gray bars) and eared doves (represented by dark blue bars). Resilience (*x* axis) was determined if a focal bird returned to a foraging patch after being disturbed (noted as 1) and a lack of it, if the individual did not return after being perturbed (noted as 0). Number of individuals (*y* axis) illustrates the number of birds of each species that were resilient or not to the disturbance.

We found a significant positive correlation between flock size and resilience (Table 2). Birds foraging in larger flocks were more resilient to being ran toward when compared to those foraging by themselves or in smaller flocks (Figure 3). Furthermore, resilience varied among species, with ED being more resilient to disturbances than GT (Figure 4, Table 2). Also, although not statistically significant, birds tended to be more resilient when they were found in habitats with higher urbanization levels, closer to an escape cover, and when foraging on grass or concrete (Table 2).

**TABLE 2:** Coefficients and standard errors from a Generalized Linear Model (GLM) that evaluated the influence of ecological factors (fixed effects) on the resilience of individuals.

Fixed effect	Estimate	SE	*z*	*p*
Intercept	−1.15	0.6	−1.91	0.06
Distance to escape cover	−0.67	0.37	−1.81	0.07
Urbanization level	−0.13	0.26	−0.51	0.61
Flock size	**0.84**	**0.28**	**3.02**	**0.002**
Place (Grass)	−1.18	0.68	−1.76	0.08
Place (Ground)	−0.92	0.69	−1.34	0.18
Species (Great thrush)	**1.44**	**0.52**	**2.77**	**0.006**

*Note:* Bold values indicate statistical significance at α = 0.05. Reference level for the variable microhabitat was concrete and species was Great thrush. *N* = 113 corresponds to the total of individuals included in the regression. The prediction of the model with just its intercept was explained by a null deviance of 132.78 on 112 degrees of freedom and with the predictors by a residual deviance of 103.97 on 106 degrees of freedom. AIC of the model was 117.97.

### 3.2 Recovery Strategies Were Influenced by Flock Size and Varied Among Species

**FIGURE 5:**
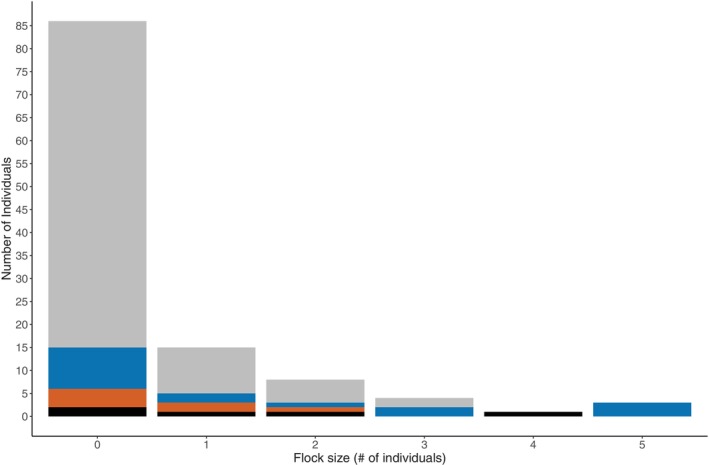
Variation in the flock size of the individuals that employed different recovery strategies: Disturbance avoidance (gray), habituation (blue), hypervigilance (orange), and wait and see (black). *N* = 113 represent the number of observations included. Note that individuals foraging on their own are in a flock size of 0.

Flock size varied significantly among individuals that employed different strategies to recover (Table 3). Specifically, birds that displayed a habituation strategy were foraging in larger flocks when compared to individuals that employed a disturbance avoidance strategy (Figure 5). Individuals tended to exhibit disturbance avoidance, hypervigilance and wait and see strategies when their habitats had a lower urbanization level. However, this trend was not statistically significant (Table 3).

**TABLE 3:** Odds ratios, confidence intervals, and *p* values from a Multinomial Logit Model for Categorical and Multinomial Responses assessing the influence of ecological factors (species and flock size) on the strategies that individuals employed to recover from a human‐induced disturbance (running).

Predictors	Recovery strategy: habituation			Recovery strategy: hypervigilance			Recovery strategy: wait and see		
	Odds ratios	CI	*p*	Odds ratios	CI	*p*	Odds ratios	CI	*p*
(Intercept)	0.05	0.02–0.16	< 0.001	0.04	0.01–0.15	**< 0.001**	0.01	0.00–0.11	**< 0.001**
Flock size	2.10	1.31–3.37	**0.002**	1.35	0.62–2.90	0.448	2.28	1.09–4.79	**0.029**
Species (Eared dove)	3.65	1.03–12.89	**0.044**	3.31	0.60–18.17	0.169	2.12	0.17–26.52	0.559
Observations	113								
Nagelkerke's *R* ^2^	0.814								

*Note:* Reference level for species was Great thrush. *N* = 113 corresponds to the total of individuals included in the regression. AIC of the model is 168.3. Bold values indicate significant results at a 95% confidence level.

**FIGURE 6:**
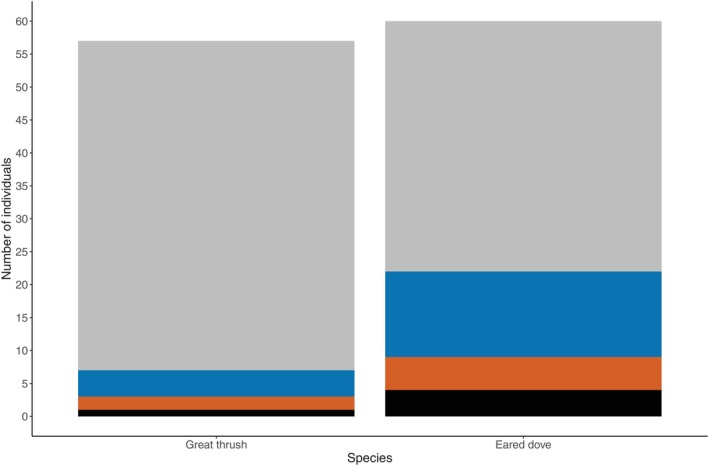
Variation in the recovery strategies that great thrushes and eared doves employed to deal with disturbances. Strategies that individuals employed to overcome disturbance were disturbance avoidance (gray), habituation (blue), hypervigilance (orange), and wait and see (black). *N* = 113 represents number of observations included.

We found that species employed different strategies to recover from disturbance (Table 3, Figure 6). Although both ED and GT exhibited disturbance avoidance as the main recovery strategy, GT used it more frequently (Table S1, Figure 6). Additionally, ED displayed habituation, hypervigilance, and waiting more frequently than GT (Table S1, Figure 6).

## 4 Discussion

Our results provide support for our prediction that the resilience of urban dwellers to human‐induced disturbances and the recovery strategies were influenced by flock size and differed between species. Individuals foraging in larger flocks were more resilient to human disturbances and coped with them employing habituation strategies. ED were more resilient to human disturbances than GT, and although both species exhibited disturbance avoidance as their main recovery strategy, GT used it more frequently. However, most urban dwellers were not resilient to human‐induced disturbances and exhibited a disturbance avoidance strategy to cope with them rather than habituating to them. Contrary to our expectations, we did not find support for the effect of distance to the closest escape cover, microhabitat, and urbanization level on resilience or recovery strategies. Overall, our findings suggest that to cope with human‐induced disturbances, urban dwellers might employ social strategies such as flocking and that species‐specific traits such as the level of habituation to humans might promote the resilience of species while foraging.

### 4.1 Resilience and Recovery Strategies Were Affected by Flock Size

Birds foraging in larger flocks were more resilient than those foraging on smaller flocks or by themselves and employed a habituation strategy. This aligns with our initial predictions of sociality being a key predictor of predation risk. Individuals in larger flocks experience a reduced probability of predation (Cresswell 1994), which may result in a reduced perceived risk but may enhance resilience. This is consistent with previous research showing that group living mitigates the effect of disturbances (e.g., Komdeur and Ma 2021; Blumstein et al. 2023). Although the relationship between habituation strategy and flock size, to our best knowledge has not been previously studied, our results are in line with studies showing that individuals foraging in larger flocks exhibit shorter escape responses than those foraging by themselves or smaller groups (Ardila‐Villamizar et al. 2022; Nepali et al. 2024), suggesting habituation to stimuli (Lin et al. 2012). Our finding suggest that sociality is a predictor of behavioral resilience, and that this could be observed across group‐living taxa.

### 4.2 Resilience and Recovery Strategies Varied Between Species

Our findings indicate that ED generally showed greater resilience and employed disturbance avoidance strategies less frequently than GT. This variation may be driven by differences in their diet and their relationship with humans (as suggested by Basile et al. 2020). Under urban conditions, ED often consume abundant human resources, which enhances their foraging success (Echeverría and Vassallo 2008) and ultimately promotes resilience to disturbance in urban ecosystems and reduces their tendency to avoid them. In contrast, GT typically forage on less abundant natural resources such as worms or fruits (Hilty and Brown 1986), which may decrease their foraging success, limiting their resilience in urban environments and promoting their tendency to avoid disturbances. Additionally, within cities, ED benefit from human provisioning in parks and open spaces, potentially reducing their perceived risk from humans (i.e., fear reduction due to feeding as proposed by Moller and Xia 2020). GT receive less human reinforcement, which may increase their perceived risk from humans. These results suggest that in urban environments, dietary preference for human resources and human attitudes toward birds may modulate birds' resilience to disturbances and strategies exhibited to overcome them.

### 4.3 Most Urban Dwellers Were Not Resilient to Human‐Induced Disturbances

Contrary to our expectations, we found that most individuals did not return to their foraging patch after being disturbed indicating a lack of resilience. The level of disturbance experienced in a foraging patch may influence the decision to resume foraging as returning might entail escaping again and using more energy (Frid and Dill 2001). A lack of resilience may result from individuals overestimating the risk they perceived from disturbances. Overestimating the risk from human‐induced disturbances aligns with previous studies showing that little bustards exhibit more vigilance and forage less during times of the week with more human‐induced disturbances (Tarjuelo et al. 2015). Also, six Brazilian mammals shifted their activity patterns due to human recreational activities (Ewart et al. 2024) and Australian small prey animals decreased their foraging rates in response to anthropogenic disturbances (Fardell et al. 2021). Overestimating risks can be relevant in environments with unreliable stimuli, such as urban ecosystems (Smith et al. 2021) but can be detrimental and lead to ecological mismatches (Peacor et al. 2011; Smith et al. 2021). However, birds' perception of risk can be species dependent as some urban birds can underestimate risk by allowing closer human approaches, while others evoke early antipredatory responses in response to them (Samia et al. 2015; Stankowich and Blumstein 2005). Therefore, further research is needed to understand the drivers of these variation in animals' response to disturbance in urban ecosystems.

### 4.4 Disturbance Avoidance Was the Most Exhibited Recovery Strategy

Contrary to our expectations, most individuals exhibited a disturbance avoidance strategy to cope with disturbances. Choosing to abandon a patch after being disturbed rather than resuming foraging may suggest that individuals prefer to forage in undisturbed patches, as it reduces the probability of being disturbed again and decreases the energetic costs of scaping (Dwinnell et al. 2019). Indeed, previous studies reported that ungulates tend to forage more frequently in undisturbed areas of their habitats than disturbed ones (Dwinnell et al. 2019) and that the quantity of birds foraging in a patch decreased as disturbance increased (Fernández‐Juricic 2000; Fitzpatrick and Bouchez 2010; Navedo et al. 2019).

Urban dwellers can also exhibit a disturbance avoidance strategy as a result of being highly efficient in extracting foraging resources or because urban patches are of low quality (Shochat et al. 2004; Lerman et al. 2012; Aronson et al. 2017). The probability of leaving a food patch is impacted by the profits that an individual can acquire in the patch, which is related to food availability (Flaherty et al. 2010; Arcis and Desor 2003; Yang et al. 2015). Food availability is usually limited in urban ecosystems (Aronson et al. 2017) thus suggesting that limited food availability in urban ecosystems influences the decision to not return to a patch in which they were disturbed. Alternatively, individuals may not return to the place they were disturbed at because they may have fulfilled their energy needs before being perturbed. This is consistent with previous studies finding that urban dwellers are more efficient foraging than their counterparts in natural ecosystems (Lerman et al. 2012; Shochat et al. 2004). Therefore, our results suggest that recovery strategies are influenced by a diversity of factors including environmental aspects, foraging preferences, or behavioral strategies.

### 4.5 Urbanization Level, Distance to Nearest Escape Cover and Microhabitat Did Not Influence Resilience or the Recovery Strategies of Urban Dwellers

Although urbanization level and distance to nearest escape cover are important predictors of risk perception in birds (Tryjanowski et al. 2016; Tätte et al. 2018), we did not find a relationship among them and behavioral resilience or recovery strategies. These could be due to individuals experiencing a similar intensity of disturbances across study sites (Mean_noise_ = 56.17 dB, Variance_noise_ = 50.35. Mean_pedestrian_ = 7.99 pedestrians/min, Variance_pedestrian_ = 5.39. Mean_domestic animals_ = 1.38 animals/min, Variance_domestic animal_ = 0.95) that may have masked the effects of urbanization. Consequently, despite experiencing varying levels of urbanization, birds in these areas are regularly exposed to disturbances with a similar intensity, which may have led to comparable foraging resilience. Additionally, distance to closest escape cover not being a predictor of resilience and recovery strategies could be a result of not all refuges providing the same perception of safety for birds, suggesting that their quality might be more relevant than the distance to them (as suggested by Tätte et al. 2018). This aligns with a study reporting that birds opted to escape to refuges at greater heights such as trees rather than closer available escape covers (Morelli et al. 2022). However, studies that explicitly assess the influence of refuge quality in the resilience and recovery strategies of urban dwellers are needed to confirm this hypothesis. Lastly, a larger extent of vegetation cover could enhance the quality of a patch (Molokwu‐Odozi et al. 2007) and thus, the resilience of birds that forage on them. However, such differences in vegetation cover might not be strong enough to influence the decision to resume foraging in patches with a higher vegetation cover (grass) than in those without (soil and concrete).

### 4.6 Possible Influence of Latitude on Resilience and Recovery Strategies

The effects of sociality on resilience and recovery strategies may vary along latitudinal gradients due to shifts in avian community composition. Avian communities change across latitudes (as seen in Leveau et al. 2017), potentially influencing the prevalence of social species within a community and its resilience. Moreover, tropical animals tend to be more territorial (Stutchbury and Morton 2008), which may result in highly resilient communities dominated by territorial individuals closely tied to specific areas (i.e., The habitat connectivity hypothesis: Radvan et al. 2023). Thus, the resilience of a community to disturbance could depend on the life history traits of the species, specifically on the proportion of social and territorial species within the community. However, further studies are needed to confirm this.

Moreover, factors such as urbanization level—which did not influence the resilience of our two study species—may have different effects when assessed under a latitudinal gradient. Urbanization intensity tends to be more intense at lower latitudes (Smit 2021), potentially resulting birds in those regions being more resilient to disturbances. However, areas at higher latitudes also have a longer urbanization history, which may have result in their animals having a greater experience with human‐induced disturbances and thus increased resilience to non‐domestic predators (as suggested by Johnson and Munshi‐South 2017). This variation in urbanization intensity can also result in differences among the intensity of local urbanization gradients (Zhang et al. 2021) and thus, variation in resilience within the same city. Additionally, factors we did not fully consider such as predation pressure by non‐domestic, can affect the escape responses of animals (Frid and Dill 2001) and, consequently, their resilience and recovery strategies. Since predation pressure tends to be higher at northern latitudes, birds in these regions might exhibit lower resilience to disturbances to minimize predation risk (suggested by Díaz et al. 2013). However, these hypotheses need empirical testing.

### 4.7 Recommendations for Future Studies

Resilience has conventionally been used to understand an ecosystem's ability to recover from an ecological disturbance. However, novel approaches have proposed to use this approach to assess individual‐level responses to environmental disturbances, including human‐induced. This approach may provide insights about species adaptation to disturbances in human‐intervened environments. Nonetheless, assessing resilience with a short‐term behavioral metric can have some limitations. First, resilience has a multidimensional nature of resilience not only encompass by immediate behavioral responses but also long‐term adaptations and physiological coping mechanisms (as suggested by Reed et al. 2024). Hence, future research should consider a multidimensional perspective that includes behavioral observations as well as physiological indicators and fitness outcomes (e.g., survival or reproduction). Moreover, intrinsic traits such as age, sex and previous experiences with humans may influence resilience and recovery strategies thus, and thus should also be accounted for (Fallon et al. 2020). Moreover, a reduced FID post‐disturbance might reflect tolerance or learning cognitive processes to the disturbance, not necessarily resilience (Lin et al. 2012). Adopting more integrative approach can provide a more comprehensive understanding of how animals cope, recover and adapt to recurring disturbances in human‐altered environments.

Lastly, although we aimed to minimize variation between pre‐ and post‐disturbance starting distances, we were unable to standardize them because trials were conducted in public spaces where approaching birds from fixed distances was not feasible. We believe this had minimal impact on our results; however, variation in starting distance could have potentially influenced FID estimates and, consequently, confounded interpretations of recovery strategies. Therefore, future studies should strive to standardize or statistically control pre‐ and post‐disturbance starting distances to improve the accuracy of behavioral comparisons.

## 5 Conclusion

Our study provided insights into the factors influencing resilience and the strategies that individuals use to cope with human‐induced disturbances in urban ecosystems. By focusing on two common urban bird species, we showed that species and social context (i.e., flock size) may influence how individuals recover from disturbances. ED foraging in larger flocks were generally more resilient and more likely to habituate to disturbances (i.e., reduce their escape response) compared to GT foraging in smaller flocks or alone. We also showed that despite daily exposure to human disturbances most urban dwellers did not exhibit resilience after being disturbed. This suggests that these individuals may overestimate the risk from disturbance and choose not to resume foraging to avoid costs associated to escaping repeatedly thus, maximizing their benefits. Birds responded to human disturbance (running) mainly by avoiding it (disturbance avoidance), and to a lesser extent by reducing their response (habituation), increasing their vigilance (hypervigilance) or moving and waiting for the disturbance to pass (wait and see). While these findings offer valuable observations, they are based on a limited number of species and contexts. As such, broader taxonomic and ecological studies are needed to fully understand the generality of these behavioral patterns and the factors shaping resilience in urban wildlife.

## Author Contributions

**Melissa Ardila‐Villamizar:** conceptualization (lead), data curation (lead), formal analysis (lead), investigation (lead), methodology (lead), project administration (lead), writing – original draft (lead). **Daniela T. Sandoval:** investigation (supporting), methodology (supporting), writing – review and editing (supporting). **Adriana A. Maldonado‐Chaparro:** conceptualization (supporting), funding acquisition (lead), investigation (equal), methodology (equal), writing – review and editing (lead).

## Ethics Statement

This study did not require ethical permissions from the University committee, as it did not involve direct manipulation of the animals.

## Conflicts of Interest

The authors declare no conflicts of interest.

## Supporting information


Appendix S1.

## Data Availability

The code and data to replicate the results of this study can be found in Maldonado‐Chaparro (2025).
