# Supplementary material for: Birds of a Feather Resist Together: Sociality and Species Predict the Resilience and Recovery Strategies of Two Neotropical Birds
Source: Ecol Evol. 2025 Jun 27;15(7):e71668. doi: 10.1002/ece3.71668 (PMC12204763; doi:10.1002/ece3.71668)
Supplement: Supplementary file 1 — Appendix S1. [file ECE3-15-e71668-s001.docx]

# Supporting information

# Supplementary Figures and Tables

## Supplementary Figures

**
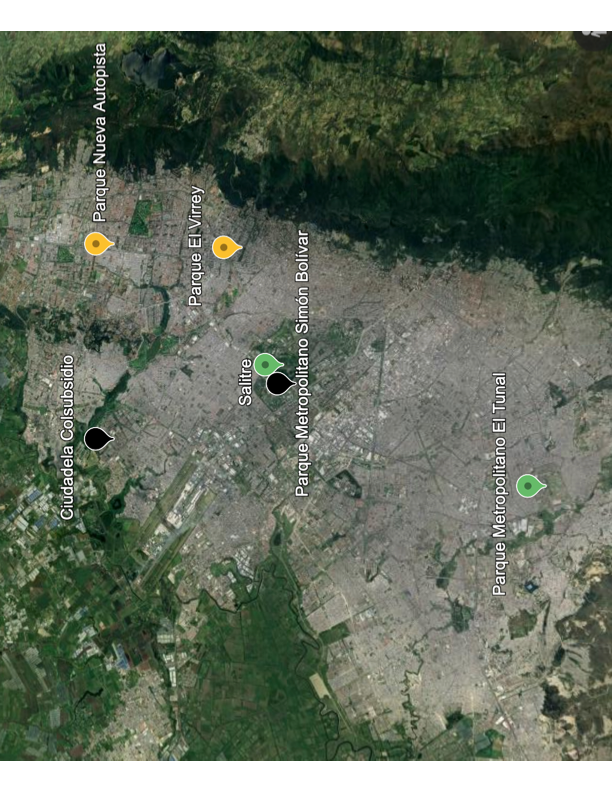
**

***Figure S1.*** Study sites’ location within Bogota. The map shows the location of 6 study sites from three categories. Each study category had two replicates. Residential areas are indicated by a black bubble, Metropolitan parks by a green bubble and Zonal parks by a yellow bubble. Site categories were selected based on their level of urbanization. The image was recovered from Google Earth. See the specific places in which each trial was conducted in <https://earth.google.com/earth/d/1IlyiRlhxshexZnCUJTuk6Q0u9wEh5YW2?usp=sharing>

And where environmental factors were recorded in <https://earth.google.com/earth/d/1QXi21FieruEX0iDDb_P9reGp8iwH_174?usp=sharing>

## Supplementary tables

***Table S1.*** Number of individuals that employed each of the recovery strategies per species.

| Species | Disturbance avoidance | Habituation | Hypervigilance | Wait and see |
| --- | --- | --- | --- | --- |
| Eared doves | 24/53 | 13/17 | 5/7 | 2/3 |
| Great thrushes | 29/53 | 4/17 | 2/7 | 1/3 |
